# Aluminium–silicon interactions in higher plants: an update

**DOI:** 10.1093/jxb/eraa024

**Published:** 2020-01-17

**Authors:** Martin J Hodson, David E Evans

**Affiliations:** 1 Department of Biological and Medical Sciences, Faculty of Health and Life Sciences, Oxford Brookes University, Headington, Oxford, UK; 2 Bielefeld University, Germany

**Keywords:** Aluminium toxicity, co-deposition, hydroxyaluminosilicates, silicon, phytolith, X-ray microanalysis

## Abstract

Aluminium (Al) and silicon (Si) are abundant in soils, but their availability for plant uptake is limited by low solubility. However, Al toxicity is a major problem in naturally occurring acid soils and in soils affected by acidic precipitation. When, in 1995, we reviewed this topic for the *Journal of Experimental Botany*, it was clear that under certain circumstances soluble Si could ameliorate the toxic effects of Al, an effect mirrored in organisms beyond the plant kingdom. In the 25 years since our review, it has become evident that the amelioration phenomenon occurs in the root apoplast, with the formation of hydroxyaluminosilicates being part of the mechanism. A much better knowledge of the molecular basis for Si and Al uptake by plants and of Al toxicity mechanisms has been developed. However, relating this work to amelioration by Si is at an early stage. It is now clear that co-deposition of Al and Si in phytoliths is a fairly common phenomenon in the plant kingdom, and this may be important in detoxification of Al. Relatively little work on Al–Si interactions in field situations has been done in the last 25 years, and this is a key area for future development.

## Teaser

We document 25 years of research concerning aluminium–silicon interactions in plants. This includes: the amelioration of aluminium toxicity by silicon; the transport of aluminium and silicon; and co-deposition in phytoliths.

## Introduction

Silicon (Si) is, at 27.7%, the second most abundant element after oxygen in the Earth’s crust, whilst aluminium (Al) is the third most abundant at 8.2% (Exley, 1998). In many soils, aluminosilicates form a very significant component of the solid mineral matter. However, both Si and Al have relatively low solubility, with Si at around a maximum of 2000 µmol l^−1^, and the solubility of Al is highly affected by pH. The uptake of both is dependent on molecular form. Uptake of Si is as Si(OH)_4_ in aqueous solution (Raven, 1983; Epstein, 1994). It is understood to travel through the plant as Si(OH)_4_ in the transpiration flow and until deposited (usually as SiO_2_: Casey *et al.*, 2003; Mitani *et al.*, 2005; Ma and Yamaji, 2015; Coskun *et al.*, 2019). Silica is deposited in the lumen, cell wall, and intercellular spaces, and the deposits are known as phytoliths. Al uptake is strongly pH dependent and is predominantly as Al^3+^ which is only available below pH 4.5 (Marion *et al.*, 1976).

There is a wide variation in the extent to which different species take up Si. It has been suggested that Si accumulators should be defined as those species whose Si content is >1.0% and where the Si/Ca ratio is >1.0 (Ma and Takahashi, 2002). Hodson *et al.* (2005) conducted a meta-analysis of data for Si content in leaves and non-woody material in 735 species. The horsetails and non-vascular plant species accumulated more Si than the ferns, gymnosperms, and angiosperms. Within the angiosperms, dicots tended to be low Si accumulators, while the commelinid monocot orders Poales (including the cereals and grasses) and Arecales accumulated substantially more Si than other monocots. In the heavy accumulators, some tissues and organs (e.g. the inflorescence bracts of the grasses) accumulate Si to very high levels. For most plants, Si is usually seen as a beneficial element, rather than essential, but it has important roles in defence against pathogens and grazers, and in decreasing abiotic stresses.

As with Si, there is a wide range of uptake of Al. For most plants, Al is toxic, and it is an important factor in acid soils around the world, where it often seriously limits plant growth. In most plants, it is excluded from the root (for a recent review, see Bojorquez-Quninal et al., 2017). There are also a small number of Al accumulators, for which Al is beneficial (e.g. tea, *Camellia sinensis*; Matsumoto *et al.*, 1976). The exclusion of Al from the root is most frequently achieved by the secretion of organic acids through transporters induced in the presence of Al. The Al accumulator species transport Al to the shoot, where it is chelated, often with organic acids (Ma et al., 1997*a*, *b*).

We last reviewed this topic 25 years ago (Hodson and Evans, 1995), and it seems timely to bring it up to date now. Considerable progress has been made during this time. We have a much better knowledge of the solution chemistry underlying Al–Si interactions, and of the mechanism whereby Al toxicity is ameliorated in root systems. We now have a good understanding of the molecular basis of Al and Si transport, and of co-deposition within the plant. In this review we will consider first the state of knowledge of the field when we last reviewed it in 1995, and then the advances made in the study of Al–Si interactions and evidence for molecular mechanisms. Finally we will re-evaluate the significance of this for agriculture and understanding of plant responses to Al in soils.

## What was known in 1995

When we reviewed the topic in 1995, the amelioration of Al toxicity by Si had already been investigated in eight species, all in hydroponic solutions. Of these, half of the investigations (for rice, wheat, cotton, and pea) showed little or no amelioration, while the other half (for sorghum, barley, teosinte, and soybean) showed a marked effect. At the time it was unclear why this was the case, and there seemed to be both plant species effects and solution effects. It was highly uncertain whether amelioration effects were to be found entirely in bulk solutions, within the plants, or a combination of the two.

In 1995 it was very well known that Al was more available for plant uptake, and more toxic, at low pH. It was not then clear, however, that pH also affected the interactions between Al and Si in solution, impacting plant growth. At that time, Baylis *et al.* (1994) had only recently published their work on amelioration of Al toxicity by Si in soybean. They showed that higher concentrations of Si were required for amelioration at lower pH, and concluded that, ‘These results support the hypothesis that the pH-dependent affinity of Si for Al in dilute solutions, and the consequent formation of sub-colloidal inert hydroxyaluminosilicate species, is the basis for the alleviation of Al toxicity by Si.’ Although we were aware that pH had an effect on hydroxyaluminosilicate (HAS) formation, it was only after this work (Baylis *et al.*, 1994) that plant scientists began to investigate the phenomenon. Birchall, Exley, and their colleagues had already investigated the chemistry of Al–Si interactions in solution, and particularly the formation of HAS (Exley and Birchall, 1992, 1993). HAS formation involved the inhibition of aluminium hydroxide nucleation. This resulted from silicic acid exchanging with hydroxylated Al at growth sites on the lattices of aluminium hydroxide. The effect depended on solution pH and the concentration of silicic acid.

It was already clear that there were several distinct groups of plants with respect to Al–Si interactions (Hodson and Evans, 1995). The grasses and cereals transported large amounts of Si to the shoot (see ‘Si transport mechanisms’ below), but mostly prevented Al transport beyond the root. Thus, most of the interest in Al–Si interactions in these species was focused on the roots. Most of the dicots excluded both elements from the shoots, and there was already some evidence (Baylis *et al.*, 1994) that Si could ameliorate Al toxicity under some conditions in these plants. Again this pointed to the roots as the main site of any amelioration effect. The phenomenon of Al hyperaccumulation in the shoot was already well recognized in some dicots and ferns, but it appeared that these plants largely excluded Si from the shoot, and that Al tolerance in the shoot mainly involved chelation with organic acids. It seemed that the conifers could transport moderate amounts of both Al and Si to the shoot. The first microanalytical study that demonstrated Al–Si co-deposition in conifer needles was that of Godde *et al.* (1988). They investigated spruce that was suffering from dieback in Germany, and found that Al and Si were co-located in the transfusion tissue of the needles. By 1995 it was already evident that there was no relationship between the uptake of Si and tolerance to Al, and that a variety of mechanisms and processes were involved.

After 1995 we chose to work on Al–Si interactions in the cereals and the conifers. Both are economically important groups, and they appeared to cope with Al toxicity, and to use Si in amelioration of the toxicity, in somewhat different ways. Other scientists joined us in these investigations, and yet others continued the work on Al hyperaccumulators. Progress since 1995 has focused on three main areas: the amelioration of Al toxicity by Si in root systems; Al and Si transport at the cellular level; and the co-deposition of Al and Si in phytoliths.

## The amelioration of Al toxicity by Si in root systems

Since our 1995 paper there have been many reports of Al toxicity being ameliorated by Si (e.g. Hammond *et al.*, 1995; Corrales *et al.*, 1997; Hara *et al.*, 1999; Liang *et al.*, 2001; Singh *et al.*, 2011; Pontigo *et al.*, 2017) and we will not catalogue these in detail here. Rather we will highlight work that elucidates the mechanism(s) behind the amelioration effect.

### The crucial effect of pH

It was soon found that even relatively small changes in solution pH can have a very marked effect on amelioration of Al toxicity by Si. Cocker *et al.* (1997), working on wheat, observed amelioration at pH 4.6, but not at 4.2 (with 100 µmol l^−1^ Al and 2000 µmol l^−1^ Si). Similarly, Ryder *et al.* (2003) found that Si (1000 µmol l^−1^ or 2000 µmol l^−1^) did not ameliorate toxic effects of Al (with 100 µmol l^−1^) on root growth of spruce at pH 4.00, 4.25, and 4.50, while apparently complete amelioration was found at pH 4.75 and 5.00. It became obvious that the early papers that reported no amelioration by Si were almost certainly using a solution pH that was too low for the effect to be observed. The importance of pH change of culture solutions during plant growth experiments was also recognized, with later work mostly being carried out in highly controlled flowing culture systems.

### Solution chemistry

It is now recognized that the chemistry of the interaction between Al and Si in solution is complex, and more complex than we had thought 25 years ago. Computer modelling of speciation has helped, and the constants used have been continually improved. Ryder *et al.* (2003) used an equilibrium speciation model (EQ3NR) to predict the behaviour of Al and Si in growth solutions over the pH range 4.0–5.0. As might be expected, addition of Si (1000 µmol l^−1^) to Al (100 µmol l^−1^) solutions caused a decrease in Al^3+^. At pH 4.00, Al^3+^ decreased from 92.4% to 83.3%, and at pH 5.00 the fall was from 54.6% to 17.7%. The decline in Al^3+^ was considered to be due to the formation of HAS species. One of the complexities in using such equilibrium speciation models is that they assume that solutions have come to equilibrium, and this may not always be the case. The most detailed recent explanation of the formation of HAS was provided by Beardmore *et al.* (2016). The authors used computational chemistry to recognize and describe the significant reaction steps. Density-functional theory combined with solvent continuum models were used to confirm that the reactants were an aluminium hydroxide dimer and silicic acid. The reaction products were two HAS species, HAS_A_ and HAS_B_. According to Beardmore *et al.* (2016), HAS_A_ dominates in solutions where the concentration of Si(OH)_4_ is less than or equal to the total Al. However, HAS_B_ is dominant when the concentration of Si(OH)_4_ is at least double that of Al. This means that we would expect HAS_B_ to be the dominant species in almost all of the experiments conducted on plants, as in nearly all cases Si exceeds Al by a factor of 10. HAS_A_ and HAS_B_ are then precursors of much more insoluble HAS species.

### A solution effect or *in planta*?

It is now certain that at least some of the ameliorative effects of Si on Al toxicity occur in solution, with the formation of non-toxic HAS species. However, evidence has accumulated since 1995 that part of the amelioration occurs within the plant (‘*in planta*’). Later we will consider cases where Al is co-deposited with Si as a solid, decreasing the availability of Al within the plant. Here we will only look at situations where there is no obvious co-deposition, mostly concerning experimental work on root systems.

Corrales *et al.* (1997), working on maize, showed that pre-treatment of roots with 1000 µmol l^−1^ Si markedly increased growth of plants that were then exposed to Al (using a range of concentrations up to 100 µmol l^−1^). Obviously, this effect could not be due to HAS formation in the bulk solutions as Al and Si were not present in the solutions at the same time. The authors showed that the presence of Si decreased the uptake of Al into the root, and suggested that this was the reason for the better growth in the plants pre-treated with Si. A second example where solution effects could be ruled out was provided by the work of Cocker *et al.* (1998a) who studied the Al-ultrasensitive wheat cultivar, Scout 66. A low concentration of 1.5 µmol l^−1^ Al inhibited the root growth, but amelioration was observed with only 5 µmol l^−1^ Si. These low concentrations of Al and Si are well below those where the formation of HAS species has been observed. Again this is highly suggestive that in this case *in planta* effects are involved.

Cocker *et al.* (1998a) also worked on the Atlas 66 cultivar of wheat, where Al-induced malate exudation from roots was already known to be involved in Al tolerance. It was found that 2000 µmol l^−1^ Si ameliorated the toxicity of Al (100 µmol l^−1^) in this cultivar at pH 4.6. Under these conditions, malate exudation was very similar to that found in plants treated with just 100 µmol l^−1^ Al. However, addition of citrate, a well-known chelator of Al, decreased malate exudation at 5–40 µmol l^−1^ and completely inhibited it at 100 µmol l^−1^. These findings strongly indicate that the formation of HAS in the bulk solution was not the reason for the amelioration of Al toxicity, and that this must take place *in planta*. We were then able to put together all of the available data and to form a hypothesis that the root cell walls were the main sites of Al detoxification, and that HAS species or aluminosilicates formed there as the primary mechanism (Cocker *et al.*, 1998b). A number of factors were believed to be responsible: high apoplastic pH; organic substances such as malate; and local concentrations of Al and Si on or within the cell wall.

The most detailed investigation of the amelioration phenomenon published so far was that of Wang *et al.* (2004), and this provided further confirmation of the role of HAS formation in the root cell wall. They worked on an Al-sensitive maize cultivar, Lixis, using short exposure times (25 µmol l^−1^ Al with or without 1400 µmol l^−1^ Si). Monomeric Al concentrations were not reduced by Si, implying that there were no interactions in the bulk solutions, and yet significant amelioration was observed. Greater than 85% of Al was bound within the cell wall in 1 cm root apices. Al content of root apices, which were treated with Si, were no different from those with no treatment for the apoplastic sap, the symplastic sap, and the cell wall. Confirming the results of Cocker *et al.* (1998a), Si had no effect on exudation of organic acids and phenols that were induced by Al treatment. The major effect of Al treatment was to increase accumulation of Si in the cell wall fraction. This decreased mobility of Al in the apoplast, and appeared to be the reason for the amelioration effects.

Additional evidence that HAS formation in the root cell wall was involved in the amelioration of Al toxicity was provided by Kopittke *et al.* (2017). These authors used low energy X-ray fluorescence (LEXRF) to examine Al and Si distribution in the sorghum root apex. They found that Al and Si were co-localized in the mucigel and outer apoplast of the root.

It seems clear that the hypothesis that we (Cocker *et al.*, 1998b) proposed has been confirmed by further work over nearly 20 years. *In planta* co-deposition of Al with Si and the formation of HAS within the root apoplast does appear to be the mechanism behind amelioration. Very recently, Coskun *et al.* (2019) proposed their ‘apoplastic obstruction hypothesis’, suggesting that Si in the apoplast is involved in the reduction of a wide range of abiotic and biotic stresses. Although they did not consider the topic in detail, it does appear that work on the amelioration of Al toxicity by Si in plants fits reasonably well with their ideas.

## Al and Si transport at the cellular level

One of the greatest advances since our previous review has been in the identification and characterization of transporters for Si and Al at the molecular level. The presence of membrane proteins (usually modified water channels, aquaporins) facilitates uptake and transport. Exley and Guerriero (2019) suggest these are channels, rather than active transporters (though there is a suggestion that at least one Si transporter is a proton-linked antiporter; Ma *et al.*, 2007; Coskun *et al.*, 2019; see below). They therefore suggest that directionality of transport is due to the concentration gradients of soluble Si(OH)_4_ created by water flow and the removal of Si from solution, usually as SiO_2_. As different species take up Si to different extents (see above), it would be expected that uptake would correlate with the presence and activity of transporters. Bélanger *et al.* (2016) demonstrated that Si uptake was dependent on root stock rather than transpiration rates in a soybean grafting experiment, suggesting that the characteristics of root cells were more important than the transpiration rate. Exley (2015) also noted the significance of guttation—the active exudation of aqueous solutes—which may include Si(OH)_4_, from the plant surface in some circumstances, though this may be considered as a variant of passive movement in the water flow.

### Si transport mechanisms

Si transporters to date have been characterized as influx and efflux carriers suggested to provide directional transport to regions of high accumulation. First to be identified (Ma *et al.*, 2006) was an Si influx protein, named Lsi1 (for low silicon protein 1, due to its absence in a low Si-accumulating rice), an aquaporin. Lsi1 is localized to the distal side of endodermal and exodermal cells of the root. This was followed by identification of an Si efflux protein, Lsi2 (Ma *et al.*, 2007), located in the root exodermis and endodermis, but, in contrast to Lsi1, on the proximal side of these two layers. Ma *et al.* (2007) therefore suggested that movement of Si in rice is through a transport pathway traversing the endodermis and exodermis, to the xylem and the shoot. *In silico* modelling reveals that their location at the endodermis and exodermis coupled with the presence of the impermeable Casparian strip is required for effective movement of Si to the shoot (Skurai *et al.*, 2015).

Lsi1 is a member of the membrane intrinsic [MIP (major intrinsic protein) and NIPIII (Nodulin 26-like intrinsic protein 3-1)] family of aquaporins. Specificity for Si is achieved by an hourglass-like configuration of the protein, with six transmembrane domains and two half helices projecting into a central pore. This constriction, with two NPA (asparagine, proline, and alanine) domains is close to a second constriction, the selectivity filter, which determines specificity for Si. The key amino acids for Si are glycine–serine–glycine–arginine (GSGR; Hove and Bhave, 2011). The spacing of the two NPA domains is important for transport, as this renders the pore permeable to Si(OH)_4_; high Si accumulators have a 108 amino acid separation (Deshmukh *et al.*, 2015). Lsi1 also transports methylated arsenic species (Li *et al.*, 2009). Lsi2 resembles a bacterial arsenite transporter and is thought to be an Si(OH)_4_/H^+^ antiporter, though direct evidence for this is lacking (Ma *et al.*, 2007; Coskun *et al.*, 2019). It belongs to the arsenite–antimonite (ArsB) efflux (TC 2.A.45) family. Si transport activity was demonstrated using *Xenopus* oocytes pre-loaded with Si, which showed Si efflux activity (Ma *et al.*, 2007). Pommerrenig *et al.* (2020) have recently provided evidence that the NIP family of transporters originated from bacterial arsenic efflux channels through a horizontal gene transfer followed by evolution to higher plant Si and boron transporters.

The tissue location of Si transport proteins has been shown to be important for movement to different areas of the shoot and contributes to areas of high accumulation (Yamaji *et al.*, 2015). Rice accumulates >10% Si (dry weight basis) in the husk, which protects the grain from biotic and abiotic stress. In addition to Lsi1 and Lsi2, Lsi6 is a plasma membrane-localized Si transporter expressed in roots and shoots, and is located at nodes involved in Si distribution from xylem to leaf tissue (Yamaji *et al.*, 2008). In these and subsequent experiments, Yamaji *et al.* (2015) observed that Lsi2 is polarly localized under bundle sheath cells around enlarged vascular bundles (VBs). Lsi6 is localized in a xylem transfer cell layer adjacent to this; Lsi3 is in the parenchyma between VBs. Knockout of these two reduces Si in panicles and increases it in the flag leaf. Yamaji *et al.* (2015) modelled this to show that an apoplastic barrier at the bundle sheath and development of the enlarged VBs in the node are needed for Si hyperaccumulation in the husk.

While rice Si transport is the best studied system, Si transporters have been identified in a number of other species based on sequence homology and, in some instances, function. These are summarized in Table 1. They include Si influx transporters in wheat, *Triticum aestivum*, TaLsi1 (Montpetit *et al.*, 2012); barley, HvLsi1 (Chiba *et al.*, 2009; Mitani *et al.*, 2009), and Si influx and efflux channels in cucumber, CmLsi1 and CmLsi3 (Mitani *et al.*, 2011); maize, NIP2-1 and ZmLsi2 (Mitani *et al.*, 2009), perennial ryegrass, *Lolium perenne*, Lsi1 and Lsi2; (Pontigo *et al.*, 2017), and a barley homologue of Lsi6 involved in Si distribution, HvSi6 (Yamaji *et al.*, 2012). Channels predicted to allow Si movement have also been identified in castor bean, wild soybean, mung bean, date palm, red clover, pearl millet, sorghum, grape vine, and barrel medic (see Supplementary Table S1 at *JXB* online). While maize and barley show similar Si transporters to rice, the work of Mitani *et al.* (2009) suggests a different transport mechanism, as the Lsi2 homologue is located at the endodermis but immunostaining does not reveal the polarity exhibited by Lsi1 and Lsi2 in rice. The importance of Lsi2 in transport, however, is demonstrated by a correlation between expression levels and Si uptake and its ability to recover uptake in a low Si rice mutant. Studies on the Si influx transporter in two cultivars of pumpkin (*Cucurbita sativus*), one a high Si accumulator, the other a low accumulator, reveal that a change of one amino acid—Pro242 to lysine—results in loss of Si accumulation and relocation of the transporter from the plasma membrane to the endoplasmic reticulum (Mitani *et al.*, 2011). Sun *et al.* (2019), in a study of tomato root Si transporters, demonstrate that while there are two Si transporter homologues present (SILsi1, a homologue of the rice LSi1 influx transporter; and SILsi2, a homologue of the rice LSi2 efflux transporter), only SILsi1 is active. They suggest that the absence of active SILsi2 explains the low levels of Si accumulation in this species.

**Table 1.** Plant Si transporters in plant membranes with supporting references (Uniprot Consortium, 2019)

Entry	Protein names	Gene names	Organism	Length	Reference
Q6Z2T3	Aquaporin NIP2-1 (Low silicon protein 1) (NOD26-like intrinsic protein 2-1) (OsNIP2;1) (Silicon influx transporter LSI1)	NIP2-1 LSI1 SIIT1 Os02g0745100 LOC_ Os02g51110 OJ1118_ G04.16 OJ1734_E02.43 OsJ_008085	*Oryza sativa* subsp. *japonica* (rice)	298	Ma *et al.* (2006)
Q10SY9	Silicon efflux transporter LSI2 (Low silicon protein 2)	LSI2 SIET1 Os03g0107300 LOC_Os03g01700 OJ1384D03.1 OsJ_09099	*Oryza sativa* subsp. *japonica* (rice)	472	Ma *et al.* (2007)
Q67WJ8	Aquaporin NIP2-2 (Low silicon protein 6) (NOD26-like intrinsic protein 2-2) (OsNIP2;2)	NIP2-2 LSI6 Os06g0228200 LOC_Os06g12310 OsJ_019836 P0425F05.28-1	*Oryza sativa* subsp. *japonica* (rice)	298	Yamaji *et al.* (2008)
Q9AV23	Silicon efflux transporter LSI3 (Low silicon protein 3)	LSI3 Os10g0547500 LOC_Os10g39980 OSJNBa0001O14.19	*Oryza sativa* subsp. *japonica* (rice)	485	Yamaji *et al.* (2015)
G0WXH5	Silicon transporter protein	TaLsi1	*Triticum aestivum* (wheat)	295	Montpetit *et al.* (2012)
B9X078	NIP2;1 (NOD26-like intrinsic protein) (Silicon transporter)	HvLsi1 HvNIP2;2 NIP2;1	*Hordeum vulgare* (barley)	295	Chiba *et al.* (2009)
Q19KC1	Aquaporin NIP2-1 (NOD26-like intrinsic protein 2-1) (ZmNIP2-1) (ZmNIP2;1)	NIP2-1 LSI1	*Zea mays* (maize)	295	Mitani *et al.* (2009)
C6KYS1	Silicon transporter	HvLsi6	*Hordeum vulgare* (barley)	300	Yamaji *et al.* (2012)
F1SX51	Silicon transporter 1	CmLsi1(B-)	*Cucurbita moschata*	288	Mitani *et al.* (2011)
C7G3B4	Silicon transporter	ZmLsi2 100502546 ZEAMMB73_ Zm00001d027305	Zea mays (maize)	477	Mitani *et al.* (2009)
F1SX50	Silicon transporter 1	CmLsi1(B+)	*Cucurbita moschata*	288	Mitani *et al.* (2011)
I4IY30	Aquaporin silicon transporter	EaNIP3,1	*Equisetum arvense* (field horsetail) (common horsetail)	248	Grégoire *et al.* (2012)
I4IY32	Aquaporin silicon transporter	EaNIP3,3	*Equisetum arvense* (field horsetail) (common horsetail)	259	Grégoire *et al.* (2012)
I4IY33	Aquaporin silicon transporter	EaNIP3,4a	*Equisetum arvense* (field horsetail) (common horsetail)	260	Grégoire *et al.* (2012)
XP_008802606	Aquaporin silicon transporter	PdNIP2-1	*Phoenix dactylifera* (date palm)	290	Bokor *et al.* (2019)
XP_004240725.1	Si influx transporter (active)	SILs2-L1	*Solanum esculentum* (tomato)	528	Sun *et al.* (2019)
XP_010317628.1	Si efflux transporter (inactive)	SILs2_L2	*Solanum esculentum* (tomato)	516	Sun *et al.* (2019)

See Supplementary Table S1 for a full list including putative Si transporters identified from genome database annotation.

Horsetails are primitive plants with a very high Si content (Hodson *et al.*, 2005), that require Si to complete their life cycle (Chen and Lewin, 1969; Miyake and Takahashi, 1976) and provide evidence of a distinct family of Si transporters (Grégoire *et al.*, 2012). These are members of the NIP2 subfamily and, in place of the GSGR Ar/R filter, possess a STAR (serine–threonine–alanine–arginine) filter predicted to have a pore large enough to permit passage of silicic acid (Grégoire *et al.*, 2012). Three horsetail transporter subgroups were identified, and EaNIP3;1, EaNIP3;3, and EaNIP3;4 are all efficient Si transporters in the *Xenopus* oocyte system and showed strong expression in roots. When EaNIP3;1 was expressed in Arabidopsis, it resulted in accumulation of Si in Arabidopsis shoots. A study of date palm (Bokor *et al.*, 2019) resulted in characterization of an aquaporin Si transporter, also possessing the GSGR Ar/R selectivity filter, PdNIP2-1. Si transport was demonstrated using *Xenopus* oocytes.

### Al transport mechanisms

Al transporters have also been characterized for the first time in plants since we wrote our previous review, though this remains a much less well explored area than Si transporters and much of the progress has been in identifying transporters for compounds such as organic acids induced under Al stress. Table 2 presents a listing of proteins suggested to be Al transporters, together with associated references. Zhang *et al.* (2019) recently reviewed evidence for Al transporters in plants, and readers are directed to this review for detailed consideration; evidence for Al transport has been associated with plasma membrane and tonoplast proteins and with proteins of the ATP-binding cassette (ABC), aquaporin, and natural resistance-associated macrophage protein (Nramp) families. These include ABC partial homologues AtALS3 and AtSTAR1 in Arabidopsis with homologues OsSTAR1 and OsSTAR2 in rice (Larsen et al., 2005, 2007; Huang et al., 2009, 2012). AtALS3 encodes a single domain homologous to a bacterial ABC protein, while the two rice proteins contain the nucleotide-binding domain (OsSTAR1) and transmembrane domain (OsSTAR2) also of a bacterial-type ABC transporter (Huang *et al.*, 2009). However, when co-expressed in the oocyte transport assay system, OsSTAR1 and OsSTAR2 form a functional UDP-glucose transporter. The transporter Nrat1 (Nramp-natural resistance-associated macrophage protein aluminium transporter 1) was described by Xia *et al.* (2010) in rice and provided evidence that Nrat1 is specific for trivalent Al, localized to the plasma membrane of root tip cells other than the epidermis, and has a role in detoxifying Al in the vacuole; Nrat1 knockouts have enhanced Al sensitivity and increased cell wall Al. Providing evidence for aquaporin family transporters, Negishi et al. (2012, 2013) explored Al transport in the Al hyperaccumulator hydrangea where they identified both plasma membrane (PALT1) and tonoplast (VALT) Al transporters in the sepals. Both PALT1 and VALT are members of the aquaporin family. Expression in arabidopsis confers Al sensitivity (PALT) and tolerance (VALT), suggesting they are part of a pathway of Al detoxification in the vacuole.

**Table 2.** Putative aluminium transporters with supporting publications listed (UniProt Consortium, 2019)

Entry	Entry name	Protein names	Gene names	Organism	Length	Reference
Q9ZUT3	ALS3_ARATH	Protein ALUMINUM SENSITIVE 3 ABC family member, but lacking ATP binding cassette.	ALS3 ABCI16, At2g37330, F3G5.12	*Arabidopsis thaliana* (mouse-ear cress)	273	Larsen *et al.* (2005)
K0ITY9	K0ITY9_HYDMC	Plasma membrane aluminium transporter Aquaporin family	PALT1	*Hydrangea macrophylla* (bigleaf hydrangea) (*Viburnum macrophyllum*)	304	Negishi *et al.* (2012)
Q0WML0	AB27B_ARATH	ABC transporter B family member	ABCB27 ALS1, TAP2, At5g39040, MXF12.50	*Arabidopsis thaliana* (mouse-ear cress)	644	Larsen *et al.* (2007)
Q9FNU2	AB25B_ORYSJ	ABC transporter B family member	ABCB25 ALS1, Os03g0755100, LOC_Os03g54790, OSJNBb0081K01.19	*Oryza sativa* subsp. *japonica* (rice)	641	Verrier *et al.* (2008)
K0IVT1	K0IVT1_HYDMC	Vacuolar aluminium transporter Aquaporin family	VALT	*Hydrangea macrophylla* (bigleaf hydrangea) (*Viburnum macrophyllum*)	252	Negishi *et al.* (2012)
Q0D9V6	STAR1_ORYSJ	Protein STAR1 Partial ABC transporter; when expressed with STAR2 transports UDP glucose.	STAR1 ABCI12, ALS1, Os06g0695800, LOC_ Os06g48060, P0622F03.26	*Oryza sativa* subsp. *japonica* (rice)	346	Huang *et al.* (2009)
Q5W7C1	STAR2_ORYSJ	UPF0014 membrane protein STAR2 partial ABC transporter	STAR2 Os05g0119000, LOC_ Os05g02750, P0496H07.22	*Oryza sativa* subsp. *japonica* (rice)	285	Huang *et al.* (2009)
Q6ZG85	NRAT1_ORYSJ	Metal transporter NRAT1	NRAT1 Os02g0131800, LOC_ Os02g03900, OJ1007_D04.24, OsJ_05257	*Oryza sativa* subsp. *japonica* (rice)	545	Xia *et al.* (2010)

The exclusion of Al from the root is most frequently achieved by the secretion of organic acids through transporters induced in the presence of Al. Two families of transporters have been identified, designated MATEs (multidrug and toxic compound extrusion) and ALMTs (aluminium-activated malate transporters) (Bojorquez-Quninal et al., 2017; Yang *et al.*, 2019; Zhang *et al.*, 2019). As well as preventing Al uptake, other species transport Al to the shoot, where it is chelated. In high Al accumulators, such as hydrangea, buckwheat, and tea (Ma *et al.*, 1997a; Carr *et al.*, 2003; Shen *et al.*, 2004), chelation by small organic compounds is key to detoxification; in buckwheat, this is oxalate, with Al-citrate being the mobile form in the xylem (Ma *et al.*, 1997b; Shen *et al.*, 2004). In hydrangea sepals, the chelator is delphinidin 3-glucoside and is 3-caffeoylquinic and citrate in the leaves (Ma *et al.*, 1997*a*). The chelator in tea has been shown to be catechin (Nagata *et al.*, 1992).

### Evidence for a role for Si and Al transporters in amelioration

While Si involvement in the amelioration of Al toxicity could be the result of altered transporter activity, evidence for direct effects of Si on Al transporters (and vice versa) is limited. In an examination of this topic, Pontigo *et al.* (2017) explored the effect of Si on Si and Al transport in ryegrass, using a hydroponic system. Plants were cultivated in varying concentrations of Al and Si. Al uptake was decreased in the presence of Si; and Lsi1 and Lsi2 genes were both down-regulated in roots in the presence of Al and Si, applied singly. However, application of Si to Al-treated plants resulted in an up-regulation of both transporter genes. The authors suggest that this is indicative of an increased Si requirement by the tissue in Al toxicity. Bhat *et al.* (2019) have recently reviewed the importance of Si in ameliorating heavy metal toxicity. In rice, down-regulation of heavy metal transporter genes (*OsHMA2* and *OsHMA3*), and up-regulation of Si transporter genes (*OsLSi1* and *OsLSi2*) has been observed with Si supplementation (Kim *et al.*, 2014). Similarly, the enhanced expression of *phytochelatin synthase 1* (*PCS1*) and decreased expression of the *metallothionein* gene (*MT1a*) was associated with Si supplementation in Arabidopsis under copper stress (Khandekar and Leisner, 2011). Likewise, Si up-regulates OsLsi1 and down-regulates the cadmium (Cd) transporter Nramp5 in Cd stress (Ma *et al.*, 2015). While results like these for heavy metals may shed some light on the role of transporters in amelioration, it is clear additional data are needed before their role is fully understood.

## Co-deposition of aluminium and silicon

We have seen that the formation of HAS in the root apoplast appears to be part of the mechanism whereby Al toxicity is ameliorated by Si. However, as Beardmore *et al.* (2016) point out, HAS_A_ and HAS_B_ are precursors of much more insoluble HAS species. It would therefore seem quite likely that we would find solid deposits containing both Al and Si in plants. When we wrote our previous review of this topic (Hodson and Evans, 1995), relatively little was known about the co-deposition of Al and Si in plants. We considered two methodologies for studying this: chemical analysis and microanalysis. There has been less work since that time using chemical analysis, as the difficulties in obtaining samples of phytoliths that were not contaminated with minerals from non-mineralized plant tissue have become apparent. For example, Kameník *et al.* (2013) used three different extraction methodologies to isolate phytoliths from barley: acid digestion, dry ashing, and acid digestion followed by incineration. They did not recommend just using acid digestion as it left organic matter in the phytoliths, but even the other two methods gave somewhat different results. In all cases, terrigenous elements, including Al, were enriched in the phytoliths compared with the plant material. For the rest of this discussion, we will only consider microanalytical work. In 1995 we could only locate five microanalytical investigations that had concerned this topic. We now have a much better understanding of the scale of Al and Si co-deposition, although it is still far from clear how significant this is in the amelioration of Al toxicity. We will now assess the progress that has been made in this area under four headings: roots; conifers; broad-leaved woody plants (including Al accumulators); and others.

### Roots

As we have seen, the grasses and cereals seem to largely exclude Al from their shoots, and so we would expect most of the interest in Al–Si interactions in these species to be in the roots. That does appear to be the case (Cocker *et al.*, 1998b), but these interactions seem mostly to involve soluble HAS complexes, and there have only been two cases where Al and Si co-deposition has been observed. In both sorghum (Hodson and Sangster, 1993) and wheat (Cocker *et al.*, 1997) plants grown in hydroponic culture, deposits containing Al and Si were located in the root epidermal cell walls. The authors speculated that this co-deposition may prevent some Al from penetrating further into the root cortex. As we noted above, Kopittke *et al.* (2017) used LEXRF and found that Al and Si were co-localized in the mucigel and outer apoplast in the sorghum root apex, a similar location to that observed by Hodson and Sangster (1993) for the same species. LEXRF is a more sensitive technique than X-ray microanalysis, and it is possible that it was detecting more soluble HAS in addition to deposited material.

Until recently, the only example of Al and Si co-deposition in roots that we are aware of outside the grasses was from Norway spruce (Hodson and Wilkins, 1991) where the phenomenon was observed in the cortical cell walls. One slightly unusual example of Al and Si co-deposition comes from the work of Feng *et al.* (2019) working on the root border cells of pea. They were able to produce an extracellular silica nanocoat formed by layer-by-layer self-assembly on the surface of these cells. When they were then treated with Al, the coating adsorbed Al on its surface preventing it from penetrating into the cells, and thereby decreasing toxicity. The authors suggested that this approach might be used to solve the global Al toxicity problem, but we suspect that much more research will be needed before it can be applied in field situations.

### Conifer needles

Carnelli *et al.* (2002) included five conifers in their study of Al in the phytoliths isolated from plants growing in the Valaisan Swiss Alps. All of the phytoliths from wood contained Al, and the element was also detected in the needles of three species (*Juniperus nana*, *Pinus cembra*, and *Pinus mugo*). Phytoliths containing Al were also common in woody dicots, but very rarely in grasses and other monocots. These findings led the authors to suggest that Al and Si co-deposition in phytoliths could be used as a marker for woody species in palaeoecological investigations.

Following our review in 1995, one of us (MJH) was involved in a whole series of studies investigating mineral localization in the needles of conifers. The species investigated were: white spruce (Hodson and Sangster, 1998); balsam fir (Hodson and Sangster, 1999); American larch and European larch (Sangster *et al.*, 2001); white pine (Hodson and Sangster, 1999, 2000, 2002); Douglas fir (Sangster *et al.*, 2007); and Eastern hemlock (Sangster *et al.*, 2009). These studies gave us a much better idea of the extent of Al and Si co-deposition, its locations, and the environmental factors that affect it. The co-deposition of Al and Si occurred in all the species investigated, but the phenomenon was more pronounced in white pine, Eastern hemlock, balsam fir, and the larch species, and less so in white spruce and Douglas fir. The major locations for Al and Si co-deposition in the needles were the epidermis and the transfusion tissue. Epidermal deposition was most pronounced in white spruce, balsam fir, and the larch species. Al and Si co-deposition in the transfusion tissue was noted particularly towards the tips of the needles in white pine and Eastern hemlock. It was also evident from these investigations that Al and Si co-deposition was only found when the plants were growing in acidic soils. This was most clearly shown in white pine (Table 3) where both Al and Si accumulate in the transfusion tissue near the tips of the needles, but more of both elements are present in soils with a pH below 4.2. It appears that the idea of Carnelli *et al.* (2002) that Al in phytoliths could be used as a marker for woody species is only likely to work if all of the plants are growing on acidic soils with reasonable Al availability.

**Table 3.** A comparison between microanalysis results from three sites for Al and Si in the transfusion cells of 2-year-old white pine needles

Needle zone	Al (mmol kg^–1^)			Si (mmol kg^–1^)		
	Glendon pH 6.7^*a*^	Muskoka pH 4.2	Sudbury pH 3.5–4.0	Glendon pH 6.7	Muskoka pH 4.2	Sudbury pH 3.5–4.0
**Base**	9.6	12.7	14	29.2	13	16.6
**Middle**	13.9	30.2	11.1	13.1	19.5	25
**Tip**	9.1	44.5	61.4	611	5838	4282

Data from Hodson and Sangster (1999, 2000, 2002).

^*a*^ Soil pH at 40 cm.

### Broad-leaved woody plants

As we saw above, Carnelli *et al.* (2002) included seven woody shrub species in their study of Al and Si co-deposition in phytoliths from plants growing in the Swiss Alps. In all cases, Al was detected in phytoliths from leaves and wood. Turnau *et al.* (2007) conducted a detailed investigation of metal uptake in *Erica andevalensis* growing on a highly acidic pyrite mine in southeastern Portugal. Using microanalysis they showed that both iron and Al were co-localized with Si in the epidermis and glandular hairs of the leaves. They also found the elements associated with a fungal root endosymbiont, *Hymenoschyphus ericae*. The authors suggested that the endosymbiont is involved in restricting the entry of toxic metals into the roots, while co-deposition of Al with Si in the leaf epidermis was seen as a detoxification mechanism.

In our 1995 review, we wrote that, ‘It would appear that heavy Al accumulation and heavy Si accumulation are mutually exclusive.’ This statement has proven to be largely correct, but it does now appear that some Al accumulator species potentially use Si in detoxification. The best example of this phenomenon is the Al accumulator, *Faramea marginata* (Rubiaceae), which was studied by Britez *et al.* (2002). This species is not an Si accumulator, and the authors described it as ‘no more than an intermediate or weak Si accumulator’, and it does not form phytoliths. However, Britez *et al*. presented very persuasive evidence that Al and Si form complexes, probably including HAS, in their shoot tissues. Most Al accumulators use organic ligands and form complexes with Al (see above), and this was thought to be the first example where an inorganic ligand was used for detoxification. So this appears to be a case where Al and Si co-deposition does not occur, but Si is involved in reducing the availability of Al in the shoot. More recently, Malta *et al.* (2016) investigated *Rudgea viburnoides*, another Al accumulator in the Rubiaceae, and using X-ray microanalysis found that Al and Si were co-localized in the roots, stems, and leaves, mostly in the epidermis.

Probably the most famous Al accumulator plant is tea (*Camellia sinensis*) and, according to Matsumoto *et al.* (1976), it can accumulate >3% Al in its older leaves. These authors used light microscopy and X-ray microanalysis, and located Al in the epidermal cells, but did not consider Si. It appears that there is some uncertainty with regards to Al and Si co-localization in this species. Whilst Tolrà *et al.* (2011) reported that the two elements were not co-deposited, Haruyama *et al.* (2019) found that this occurred in the leaf epidermis. The latter suggested that the reason for the different distributions was possibly due to the conditions under which the plants grew and the ages of the leaves.

### Other plants

For completeness, we should also point out that Al and Si co-localization has been observed in species beyond the Angiosperms. Liu *et al.* (2019) worked on *Dicranopteris linearis*, a fern which is an Al hyperaccumulator in China. They showed co-deposition of Si with Al and rare Earth elements (REEs). This is the one case we are aware of where a species is both an Al accumulator (up to 9660 mg kg^−1^) and an Si accumulator (up to 20 300 mg kg^−1^). *Dicranopteris linearis* also accumulates high concentrations of REEs (up to 3830 mg kg^−1^). How this species is capable of doing something that few, if any, others can do is a mystery.

Finally, Pressel *et al.* (2011) found what they considered was the first example of Al and Si co-localization in the bryophytes. Working on the thalloid liverwort, *Mizutania riccardioides*, they found that the outer part of the wall consisted of irregular blocks of dense material, containing high concentrations of Al and Si.

## What questions remain?

Having assessed 25 years of research on Al–Si interactions in plants what are the topics that still need further research?

In the key area of the amelioration of Al toxicity by Si in the root, it seems that our hypothesis stated in Cocker *et al.* (1998b) is largely correct, and the formation of HAS within the apoplast is at least a major part of the mechanism. Although there has been considerable progress in determining the molecular basis for both Al and Si transport, we have only just begun to investigate the effect of Al on Si transport or of Si on Al transport (Pontigo *et al.*, 2017). It will be some time before we can determine how important these processes are in the amelioration effect. Is the *in planta* amelioration, often observed, solely due to phenomena in the apoplast which prevent Al uptake, or is it more complex than that?

A number of studies have shown that Si has a general effect on plant responses to abiotic stress (for reviews, see Kim *et al.*, 2017; Etesami and Jeong, 2018). Application of Si increases stress tolerance by reducing the generation of reactive oxygen species (ROS). These effects were seen for a wide range of abiotic stresses, including the heavy metals Cd (Shi *et al.*, 2010), manganese (Maksimović *et al.*, 2007), and chromium (Tripathi *et al.*, 2015), but evidence for Al/Si is limited. In a study of the effect of Si nanoparticles (SNPs) on Al toxicity in maize growing in an artificial soil system, de Sousa *et al.* (2019) demonstrated (amongst other effects) that while Al uptake remained the same as in the absence of SNPs, ROS production was reduced, as was oxidative damage. Antioxidant enzymes and metabolites were enhanced in leaves and roots. In a similar way, Dorneles *et al.* (2019) showed that Si was partially able to ameliorate Al toxicity in two potato genotypes. The authors related this to increased activity of antioxidant enzymes and mitigation of Al-induced damage to membrane lipids.

One of the more disappointing aspects of our review of Al–Si interactions 25 years on is how little research on agricultural systems is recorded in the literature. In fact there have been very few investigations carried out in soil systems. Morikawa and Saigusa (2002) studied the impacts of Si fertilization on Al toxicity in barley plants growing in two andosol soils. They found that addition of silica gel and sodium metasilicate to the soils was ineffective in amelioration of Al toxicity despite the treatments causing an increase in soil Si concentration. Waste porous hydrated calcium silicate did lead to amelioration of Al toxicity but the authors considered that this was probably due to the increase in soil pH. Although the paper by Morikawa and Saigusa has, according to Google Scholar, been cited 31 times (in January 2020), none of the citations refer specifically to further experiments conducted in soil to investigate Al–Si interactions. In the study by de Sousa (2019; see above) on the effect of silicon dioxide nanoparticles on Al toxicity, amelioration but not a reduction in Al accumulation was observed. The overall effect was suggested to result from stimulation of organic exudation in the roots. It may be that the paucity of studies in this area is due to the complexities of the chemistry, particularly in soil. So at present we have definite ameliorative effects in hydroponics, but uncertainty as to whether the findings can be transferred to field situations and under what conditions. The overall beneficial effect of Si on stress and Si soil treatments on pH suggest that this is worth further exploration.

We now know much more about Al and Si co-deposition than we did, particularly in the conifers. However, it is unclear whether this is part of a detoxification mechanism or is just coincidental. Al as Al^3+^ is toxic to plant cells, and precipitation in a solid form should decrease toxicity, but we have no direct experimental evidence that proves this.

## General conclusions

Our knowledge of Al–Si interactions in plants has markedly increased in the last 25 years. We have determined a mechanism for the amelioration effect in roots that has withstood detailed investigation. This mechanism shows the importance of the apoplast in the amelioration phenomenon. We now have greatly increased knowledge of Al and Si transporters but have yet to ascertain whether these have a role in amelioration. It appears that Al and Si co-deposition is quite common in some plant groups, but its functional significance is not certain. We still have relatively little knowledge concerning the importance of amelioration effects in the field, and hopefully this will be a focus of the next 25 years of research.

## Supplementary data

Supplementary data are available at *JXB* online.

Table S1. The plant membrane proteins that have been identified as Si transporters from either publications or genome database annotation.

eraa024_suppl_Supplementary_Table_S1

## Abbreviations

ABC: ATP-binding cassette
HAS: hydroxyaluminosilicate
NIPIII: Nodulin 26-like intrinsic protein 3-1
REE: rare Earth element
VB: vascular bundle

## References

[CIT0001] Baylis AD, Gragopoulou C, Davidson KJ, Birchall JD 1994 Effects of silicon on the toxicity of aluminium to soybean. Communications in Soil Science and Plant Analysis 25, 537–546.

[CIT0002] Beardmore J, Lopez X, Mujika JI, Exley C 2016 What is the mechanism of formation of hydroxyaluminosilicates? Scientific Reports 6, 30913. 27477995 10.1038/srep30913 PMC4967899

[CIT0003] Bélanger R, Deshmukh R, Belzile F, Labbé C, Perumal A, Edwards SM 2016 Plant with increased silicon uptake. Patent No.: WO/2016/183684.

[CIT0004] Bhat JA, Shivaraj SM, Singh P, Navadagi DB, Tripathi DK, Dash PK, Solanke AU, Sonah H, Deshmukh R 2019 Role of silicon in mitigation of heavy metal stresses in crop plants. Plants 8, 71. 10.3390/plants8030071 PMC6473438 30901942

[CIT0005] Bojórquez-Quintal E, Escalante-Magaña C, Echevarría-Machado I, Martínez-Estévez M 2017 Aluminum, a friend or foe of higher plants in acid soils. Frontiers in Plant Science 8, 1767. 29075280 10.3389/fpls.2017.01767 PMC5643487

[CIT0006] Bokor B, Soukup M, Vaculík M, et al. 2019 Silicon uptake and localisation in date palm (*Phoenix dactylifera*)—a unique association with sclerenchyma. Frontiers in Plant Science 10, 988. 31456812 10.3389/fpls.2019.00988 PMC6701203

[CIT0007] Britez RM, Watanabe T, Jansen S, Reissmann CB, Osaki M 2002 The relationship between aluminium and silicon accumulation in leaves of *Faramea marginata* (Rubiaceae). New Phytologist 156, 437–444. 10.1046/j.1469-8137.2002.00531.x 33873582

[CIT0008] Carnelli AL, Madella M, Theurillat JP, Ammann B 2002 Aluminum in the opal silica reticule of phytoliths: a new tool in palaeoecological studies. American Journal of Botany 89, 346–351. 21669743 10.3732/ajb.89.2.346

[CIT0009] Carr HP, Lombi E, Küpper H, McGrath SP, Wong MH 2003 Accumulation and distribution of aluminium and other elements in tea (*Camellia sinensis*) leaves. Agronomie 23, 705–710.

[CIT0010] Casey WH, Kinrade SD, Knight CTG, Rains DW, Epstein E 2003 Aqueous silicate complexes in wheat, *Triticum aestivum* L. Plant, Cell & Environment 27, 51–54.

[CIT0011] Chen CH, Lewin J 1969 Silicon as a nutrient element for *Equisetum arvense*. Canadian Journal of Botany 47, 125–131.

[CIT0012] Chiba Y, Mitani N, Yamaji N, Ma JF 2009 HvLsi1 is a silicon influx transporter in barley. The Plant Journal 57, 810–818. 18980663 10.1111/j.1365-313X.2008.03728.x

[CIT0013] Cocker KM, Hodson MJ, Evans DE 1998 *a* The amelioration of aluminium toxicity by silicon in wheat (*Triticum aestivum* L.): malate exudation as evidence for an in planta mechanism. Planta 204, 318–323.

[CIT0014] Cocker KM, Hodson MJ, Evans DE 1998 *b* The amelioration of aluminium toxicity by silicon in higher plants: solution chemistry or an in planta mechanism? Physiologia Plantarum 104, 608–614.

[CIT0015] Cocker KM, Hodson MJ, Evans DE, Sangster AG 1997 The interaction between silicon and aluminium in *Triticum aestivum* L. (cv. Celtic). Israel Journal of Plant Sciences 45, 285–292.

[CIT0016] Corrales I, Poschenrieder C, Barceló J 1997 Influence of silicon pretreatment on aluminium toxicity in maize roots. Plant and Soil 190, 203–209.

[CIT0017] Coskun D, Deshmukh R, Sonah H, Menzies JG, Reynolds O, Ma JF, Kronzucker HJ, Bélanger RR 2019 The controversies of silicon’s role in plant biology. New Phytologist 221, 67–85. 30007071 10.1111/nph.15343

[CIT0018] Deshmukh RK, Vivancos J, Ramakrishnan G, Guérin V, Carpentier G, Sonah H, Labbé C, Isenring P, Belzile FJ, Bélanger RR 2015 A precise spacing between the NPA domains of aquaporins is essential for silicon permeability in plants. The Plant Journal 83, 489–500. 26095507 10.1111/tpj.12904

[CIT0019] de Sousa A, Saleh AM, Habeeb TH, Hassan YM, Zrieq R, Wadaan MAM, Hozzein WN, Selim S, Matos M, AbdElgawad H 2019 Silicon dioxide nanoparticles ameliorate the phytotoxic hazards of aluminum in maize grown on acidic soil. The Science of the Total Environment 693, 133636. 31377375 10.1016/j.scitotenv.2019.133636

[CIT0020] Dorneles AOS, Pereira AS, Sasso VM, Possebom G, Tarouco CP, Schorr MRW, Rossato L, Ferreira PAA, Tabaldi LA 2019 Aluminum stress tolerance in potato genotypes grown with silicon. Bragantia 78, 12–25.

[CIT0021] Epstein E 1994 The anomaly of silicon in plant biology. Proceedings of the National Academy of Sciences, USA 91, 11–17. 10.1073/pnas.91.1.11 PMC42876 11607449

[CIT0022] Etesami H, Jeong BR 2018 Silicon (Si): review and future prospects on the action mechanisms in alleviating biotic and abiotic stresses in plants. Ecotoxicology and Environmental Safety 147, 881–896. 28968941 10.1016/j.ecoenv.2017.09.063

[CIT0023] Exley C 1998 Silicon in life: a bioinorganic solution to bioorganic essentiality. Journal of Inorganic Biochemistry 69, 139–144.

[CIT0024] Exley C 2015 A possible mechanism of biological silicification in plants. Frontiers in Plant Science 6, 853. 26500676 10.3389/fpls.2015.00853 PMC4598575

[CIT0025] Exley C, Birchall JD 1992 Hydroxyaluminosilicate formation in solutions of low total aluminium concentration. Polyhedron 11, 1901–1907.

[CIT0026] Exley C, Birchall JD 1993 A mechanism of hydroxyaluminosilicate formation. Polyhedron 12, 1007–1017.

[CIT0027] Exley C, Guerriero G 2019 A reappraisal of biological silicification in plants? New Phytologist 223, 511–513. 30873615 10.1111/nph.15752

[CIT0028] Feng Y, Li X, Guo S, Chen X, Chen T, He Y, Shabala S, Yu M 2019 Extracellular silica nanocoat formed by layer-by-layer (LBL) self-assembly confers aluminum resistance in root border cells of pea (*Pisum sativum*). Journal of Nanobiotechnology 17, 53. 30992069 10.1186/s12951-019-0486-y PMC6466759

[CIT0029] Godde D, Homburg H, Methfessel S, Rosenkranz J 1988 Die Röntgenanalyse hilft beider Aufklärung individueller Waldschaden. LÖLF Mitteilungen 4, 23–27.

[CIT0030] Grégoire C, Rémus-Borel W, Vivancos J, Labbé C, Belzile F, Bélanger RR 2012 Discovery of a multigene family of aquaporin silicon transporters in the primitive plant *Equisetum arvense*. The Plant Journal 72, 320–330. 22712876 10.1111/j.1365-313X.2012.05082.x

[CIT0031] Hammond KE, Evans DE, Hodson MJ 1995 Aluminium/silicon interactions in barley (*Hordeum vulgare* L.) seedlings. Plant and Soil 173, 89–95.

[CIT0032] Hara T, Gu M-H, Koyama H 1999 Ameliorative effect of silicon on aluminium injury in the rice plant. Soil Science and Plant Nutrition 45, 929–936.

[CIT0033] Haruyama Y, Fujiwara T, Yasuda K, Saito M, Suzuki K 2019 Localization of aluminum in epidermal cells of mature tea leaves. Quantum Beam Science 3, 9.

[CIT0034] Hodson MJ, Evans DE 1995 Aluminium/silicon interactions in higher plants. Journal of Experimental Botany 46, 161–171. 10.1093/jxb/eraa024 PMC7709911 31950161

[CIT0035] Hodson MJ, Sangster AG 1993 The interaction between silicon and aluminium in *Sorghum bicolor* (L.) Moench: growth analysis and X-ray microanalysis. Annals of Botany 72, 389–400.

[CIT0036] Hodson MJ, Sangster AG 1998 Mineral deposition in the needles of white spruce [*Picea glauca* (Moench.) Voss]. Annals of Botany 82, 375–385.

[CIT0037] Hodson MJ, Sangster AG 1999 Aluminium/silicon interactions in conifers. Journal of Inorganic Biochemistry 76, 89–98.

[CIT0038] Hodson MJ, Sangster AG 2000 Aluminium localization in conifers growing on highly acidic soils in Ontario, Canada. In: International symposium on impact of potential tolerance of plants on the increased productivity under aluminum stress. Research Institute for Bioresources, Okayama University, Kurashiki, Japan. 103–106.

[CIT0039] Hodson MJ, Sangster AG 2002 X-ray microanalytical studies of mineral localization in the needles of white pine (*Pinus strobus* L.). Annals of Botany 89, 367–374. 12096796 10.1093/aob/mcf052 PMC4233867

[CIT0040] Hodson MJ, White PJ, Mead A, Broadley MR 2005 Phylogenetic variation in the silicon composition of plants. Annals of Botany 96, 1027–1046. 16176944 10.1093/aob/mci255 PMC4247092

[CIT0041] Hodson MJ, Wilkins DA 1991 Localization of aluminium in the roots of Norway spruce [*Picea abies* (L.) Karst.] inoculated with *Paxillus involutus* Fr. New Phytologist 118, 273–278. 10.1111/j.1469-8137.1991.tb00977.x 33874174

[CIT0042] Hove RM, Bhave M 2011 Plant aquaporins with non-aqua functions: deciphering the signature sequences. Plant Molecular Biology 75, 413–430. 21308399 10.1007/s11103-011-9737-5

[CIT0043] Huang CF, Yamaji N, Chen Z, Ma JF 2012 A tonoplast-localized half-size ABC transporter is required for internal detoxification of aluminum in rice. The Plant Journal 69, 857–867. 22035218 10.1111/j.1365-313X.2011.04837.x

[CIT0044] Huang CF, Yamaji N, Mitani N, Yano M, Nagamura Y, Ma JF 2009 A bacterial-type ABC transporter is involved in aluminum tolerance in rice. The Plant Cell 21, 655–667. 19244140 10.1105/tpc.108.064543 PMC2660611

[CIT0045] Kameník J, Mizera J, Řanda Z 2013 Chemical composition of plant silica phytoliths. Environmental Chemistry Letters 11, 189–195.

[CIT0046] Khandekar S, Leisner S 2011 Soluble silicon modulates expression of *Arabidopsis thaliana* genes involved in copper stress. Journal of Plant Physiology 168, 699–705. 21044806 10.1016/j.jplph.2010.09.009

[CIT0047] Kim YH, Khan AL, Kim DH, Lee SY, Kim KM, Waqas M, Jung HY, Shin JH, Kim JG, Lee IJ 2014 Silicon mitigates heavy metal stress by regulating P-type heavy metal ATPases, *Oryza sativa* low silicon genes, and endogenous phytohormones. BMC Plant Biology 14, 13. 24405887 10.1186/1471-2229-14-13 PMC3893592

[CIT0048] Kim Y-H, Khan AL, Waqas M, Lee I-J 2017 Silicon regulates antioxidant activities of crop plants under abiotic stress: a review. Frontiers in Plant Science 8, 510. 28428797 10.3389/fpls.2017.00510 PMC5382202

[CIT0049] Kopittke PM, Gianoncelli A, Kourousias G, Green K, McKenna BA 2017 Alleviation of Al toxicity by Si is associated with the formation of Al–Si complexes in root tissues of sorghum. Frontiers in Plant Science 8, 2189. 29312419 10.3389/fpls.2017.02189 PMC5742621

[CIT0050] Larsen PB, Geisler MJ, Jones CA, Williams KM, Cancel JD 2005 ALS3 encodes a phloem-localized ABC transporter-like protein that is required for aluminum tolerance in Arabidopsis. The Plant Journal 41, 353–363. 15659095 10.1111/j.1365-313X.2004.02306.x

[CIT0051] Larsen PB, Cancel J, Rounds M, Ochoa V 2007 Arabidopsis ALS1 encodes a root tip and stele localized half type ABC transporter required for root growth in an aluminum toxic environment. Planta 225, 1447–1458. 17171374 10.1007/s00425-006-0452-4

[CIT0052] Li RY, Ago Y, Liu WJ, Mitani N, Feldmann J, McGrath SP, Ma JF, Zhao FJ 2009 The rice aquaporin Lsi1 mediates uptake of methylated arsenic species. Plant Physiology 150, 2071–2080. 19542298 10.1104/pp.109.140350 PMC2719116

[CIT0053] Liang Y, Yang C, Shi H 2001 Effects of silicon on growth and mineral composition of barley grown under toxic levels of aluminum. Journal of Plant Nutrition 24, 229–243.

[CIT0054] Liu W-S, Zheng H-X, Guo MN, Liu C, Huot H, Morel JL, van der Ent A, Tang Y-T, Qiu R-L 2019 Co-deposition of silicon with rare earth elements (REEs) and aluminium in the fern *Dicranopteris linearis* from China. Plant and Soil 437, 427–437.

[CIT0055] Ma J, Cai H, He C, Zhang W, Wang L 2015 A hemicellulose-bound form of silicon inhibits cadmium ion uptake in rice (*Oryza sativa*) cells. New Phytologist 206, 1063–1074. 25645894 10.1111/nph.13276

[CIT0056] Ma JF, Hiradate S, Nomoto K, Iwashita T, Matsumoto H 1997a Internal detoxification mechanism of Al in hydrangea (identification of Al form in the leaves). Plant Physiology 113, 1033–1039. 12223659 10.1104/pp.113.4.1033 PMC158226

[CIT0057] Ma JF, Takahashi E 2002 Soil, fertilizer and plant silicon research in Japan. Amsterdam: Elsevier.

[CIT0058] Ma JF, Tamai K, Yamaji N, Mitani N, Konishi S, Katsuhara M, Ishiguro M, Murata Y, Yano M 2006 A silicon transporter in rice. Nature 440, 688–691. 16572174 10.1038/nature04590

[CIT0059] Ma JF, Yamaji N 2015 A cooperative system of silicon transport in plants. Trends in Plant Science 20, 435–442. 25983205 10.1016/j.tplants.2015.04.007

[CIT0060] Ma JF, Yamaji N, Mitani N, Tamai K, Konishi S, Fujiwara T, Katsuhara M, Yano M 2007 An efflux transporter of silicon in rice. Nature 448, 209–212. 17625566 10.1038/nature05964

[CIT0061] Ma JF, Zheng SJ, Matsumoto H 1997 *b* Detoxifying aluminium with buckwheat. Nature 390, 569–570.9403684

[CIT0062] Maksimović JD, Bogdanović J, Maksimović V, Nikolic M 2007 Silicon modulates the metabolism and utilization of phenolic compounds in cucumber (*Cucumis sativus* L.) grown at excess manganese. Journal of Plant Nutrition and Soil Science 170, 739–744.

[CIT0063] Malta PG, Arcanjo-Silva S, Ribeiro C, Campos NV, Azevedo AA 2016 *Rudgea viburnoides* (Rubiaceae) overcomes the low soil fertility of the Brazilian Cerrado and hyperaccumulates aluminum in cell walls and chloroplasts. Plant and Soil 408, 369–384.

[CIT0064] Marion GM, Hendricks DM, Dutt GR, Fuller WH 1976 Aluminum and silica solubility in soils. Soil Science 121, 76–85.

[CIT0065] Matsumoto H, Hirasawa E, Morimura S, Takahashi E 1976 Localization of aluminium in tea leaves. Plant & Cell Physiology 17, 627–631.

[CIT0066] Mitani N, Chiba Y, Yamaji N, Ma JF 2009 Identification and characterization of maize and barley Lsi2-like silicon efflux transporters reveals a distinct silicon uptake system from that in rice. The Plant Cell 21, 2133–2142. 19574435 10.1105/tpc.109.067884 PMC2729598

[CIT0067] Mitani N, Ma JF, Iwashita T 2005 Identification of the silicon form in xylem sap of rice (*Oryza sativa* L.). Plant & Cell Physiology 46, 279–283. 15695469 10.1093/pcp/pci018

[CIT0068] Mitani N, Yamaji N, Ago Y, Iwasaki K, Ma JF 2011 Isolation and functional characterization of an influx silicon transporter in two pumpkin cultivars contrasting in silicon accumulation. The Plant Journal 66, 231–240. 21205032 10.1111/j.1365-313X.2011.04483.x

[CIT0069] Miyake Y, Takahashi E 1976 Effect silicon on the growth of silicophile plant. Comparative studies on the silica nutrition in plants (Part 9). Journal of the Science of Soil and Manure, Japan 47, 375–382.

[CIT0070] Montpetit J, Vivancos J, Mitani-Ueno N, Yamaji N, Rémus-Borel W, Belzile F, Ma JF, Bélanger RR 2012 Cloning, functional characterization and heterologous expression of *TaLsi1*, a wheat silicon transporter gene. Plant Molecular Biology 79, 35–46. 22351076 10.1007/s11103-012-9892-3

[CIT0071] Morikawa CK, Saigusa M 2002 Si amelioration of Al toxicity in barley (*Hordeum vulgare* L.) growing in two Andosols. Plant and Soil 240, 161–168.

[CIT0072] Nagata T, Hayatsu M, Kosuge N 1992 Identification of aluminium forms in tea leaves by ^27^Al NMR. Phytochemistry 31, 1215–1218.

[CIT0073] Negishi T, Oshima K, Hattori M, Kanai M, Mano S, Nishimura M, Yoshida K 2012 Tonoplast- and plasma membrane-localized aquaporin-family transporters in blue hydrangea sepals of aluminum hyperaccumulating plant. PLoS One 7, e43189. 22952644 10.1371/journal.pone.0043189 PMC3430636

[CIT0074] Negishi T, Oshima K, Hattori M, Yoshida K 2013 Plasma membrane-localized Al-transporter from blue hydrangea sepals is a member of the anion permease family. Genes to Cells 18, 341–352. 23433438 10.1111/gtc.12041

[CIT0075] Pommerrenig B, Diehn TA, Bernhardt N, et al. 2020 Functional evolution of nodulin 26-like intrinsic proteins: from bacterial arsenic detoxification to plant nutrient transport. New Phytologist 225, 1383–1396. 31550387 10.1111/nph.16217

[CIT0076] Pontigo S, Godoy K, Jiménez H, Gutiérrez-Moraga A, Mora ML, Cartes P 2017 Silicon-mediated alleviation of aluminum toxicity by modulation of Al/Si uptake and antioxidant performance in ryegrass plants. Frontiers in Plant Science 8, 642. 28487719 10.3389/fpls.2017.00642 PMC5404182

[CIT0077] Pressel S, P’Ng KMY, Duckett JG 2011 An ultrastructural study of the liverwort *Mizutania riccardioides* Furuki et Iwatsuki: new insights into its systematic affinities and unique surface ornamentation. The Bryologist 114, 38–51.

[CIT0078] Raven JA 1983 The transport and function of silicon in plants. Biological Reviews 58, 179–207.

[CIT0079] Ryder M, Gérard F, Evans DE, Hodson MJ 2003 The use of root growth and modelling data to investigate amelioration of aluminium toxicity by silicon in *Picea abies* seedlings. Journal of Inorganic Biochemistry 97, 52–58. 14507460 10.1016/s0162-0134(03)00181-8

[CIT0080] Sangster AG, Hodson MJ, Huang CX 2001 X-ray microanalytical studies of mineral composition in cell walls of needle tissues of American larch [*Larix laricina* (Du Roi) K. Koch] and European larch [*L. decidua* (L.) Mill.] In: Labrecque M, ed. L’arbre 2000 The Tree. 4th International Symposium on the Tree, Montreal Botanic Garden. Montreal, Canada: Isabelle Quentin Press, 160–167.

[CIT0081] Sangster AG, Hodson MJ, Ling LEC 2009 Biomineralisation/environment interactions in conifers: illustrated by hemlock, *Tsuga canadensis* (L.) Carr. Quaternary International 193, 3–10.

[CIT0082] Sangster AG, Ling L, Gérard F, Hodson MJ 2007 X-ray microanalysis of needles from Douglas fir growing in environments of contrasting acidity. Water, Air and Soil Pollution: Focus 7, 143–149.

[CIT0083] Shen R, Iwashita T, Ma JF 2004 Form of Al changes with Al concentration in leaves of buckwheat. Journal of Experimental Botany 55, 131–136. 14645389 10.1093/jxb/erh016

[CIT0084] Shi G, Cai Q, Liu C, Wu L 2010 Silicon alleviates cadmium toxicity in peanut plants in relation to cadmium distribution and stimulation of antioxidative enzymes. Plant Growth Regulation 61, 45–52.

[CIT0085] Singh VP, Tripathi DK, Kumar D, Chauhan DK 2011 Influence of exogenous silicon addition on aluminium tolerance in rice seedlings. Biological Trace Element Research 144, 1260–1274. 21681465 10.1007/s12011-011-9118-6

[CIT0086] Skurai G, Satake A, Yamaji N, Mitani-Ueno N, Yokozawa M, Feugier FG, Ma JF 2015 In silico simulation modeling reveals the importance of the Casparian strip for efficient silicon uptake in rice roots. Plant & Cell Physiology 56, 631–639. 25673476 10.1093/pcp/pcv017

[CIT0087] Sun H, Duan Y, Mitani-Uneo N, Che J, Jia J, Liu J, Guo J, Ma JF, Gong H 2019 Tomato roots have a functional silicon influx transporter but not a functional silicon efflux transporter. New Phytologist doi:10.1111/pce.13679 10.1111/pce.13679 31724184

[CIT0088] Tolrà R, Vogel-Mikuš K, Hajiboland R, et al. 2011 Localization of aluminium in tea (*Camellia sinensis*) leaves using low energy X-ray fluorescence spectro-microscopy. Journal of Plant Research 124, 165–172. 20422247 10.1007/s10265-010-0344-3

[CIT0089] Tripathi DK, Singh VP, Prasad SM, Chauhan DK, Dubey NK 2015 Silicon nanoparticles (SiNp) alleviate chromium (VI) phytotoxicity in *Pisum sativum* (L.) seedlings. Plant Physiology and Biochemistry 96, 189–198. 26298805 10.1016/j.plaphy.2015.07.026

[CIT0090] Turnau K, Henriques FS, Anielska T, Renker C, Buscot F 2007 Metal uptake and detoxification mechanisms in *Erica andevalensis* growing in a pyrite mine tailing. Environmental and Experimental Botany 61, 117–123.

[CIT0091] UniProt Consortium 2019 UniProt: a worldwide hub of protein knowledge. Nucleic Acids Research 47, D506–515. 30395287 10.1093/nar/gky1049 PMC6323992

[CIT0092] Verrier PJ, Bird D, Burla B, et al. 2008 Plant ABC proteins—a unified nomenclature and updated inventory. Trends in Plant Science 13, 151–159. 18299247 10.1016/j.tplants.2008.02.001

[CIT0093] Wang Y, Stass A, Horst WJ 2004 Apoplastic binding of aluminum is involved in silicon-induced amelioration of aluminum toxicity in maize. Plant Physiology 136, 3762–3770. 15502015 10.1104/pp.104.045005 PMC527173

[CIT0094] Xia J, Yamaji N, Kasai T, Ma JF 2010 Plasma membrane-localized transporter for aluminum in rice. Proceedings of the National Academy of Sciences, USA 107, 18381–18385. 10.1073/pnas.1004949107 PMC2972927 20937890

[CIT0095] Yamaji N, Chiba Y, Mitani-Ueno N, Feng Ma J 2012 Functional characterization of a silicon transporter gene implicated in silicon distribution in barley. Plant Physiology 160, 1491–1497. 22992512 10.1104/pp.112.204578 PMC3490590

[CIT0096] Yamaji N, Mitatni N, Ma JF 2008 A transporter regulating silicon distribution in rice shoots. The Plant Cell 20, 1381–1389. 18515498 10.1105/tpc.108.059311 PMC2438455

[CIT0097] Yamaji N, Sakurai G, Mitani-Ueno N, Ma JF 2015 Orchestration of three transporters and distinct vascular structures in node for intervascular transfer of silicon in rice. Proceedings of the National Academy of Sciences, USA 112, 11401–11406. 10.1073/pnas.1508987112 PMC4568664 26283388

[CIT0098] Yang JL, Fan W, Zheng SJ 2019 Mechanisms and regulation of aluminum-induced secretion of organic acid anions from plant roots. Journal of Zhejiang University. Science. B 20, 513–527. 31090277 10.1631/jzus.B1900188 PMC6568218

[CIT0099] Zhang X, Long Y, Huang J, Xia J 2019 Molecular mechanisms for coping with Al toxicity in plants. International Journal of Molecular Sciences 20, 1551. 10.3390/ijms20071551 PMC6480313 30925682

