# Supplementary material for: Aluminium–silicon interactions in higher plants: an update
Source: J Exp Bot. 2020 Jan 17;71(21):6719–29. doi: 10.1093/jxb/eraa024 (PMC7709911; doi:10.1093/jxb/eraa024)
Supplement: eraa024_suppl_Supplementary_Table_S1 [file eraa024_suppl_supplementary_table_s1.pdf]

Supplementary Table S1. Plant membrane proteins identified as Si transporters either from publications or genome database annotation. The UniProt Consortium (2020)

Entry	Protein names	Gene names	Organism	Length	Reference
Q6Z2T3	Aquaporin NIP2-1 (Low silicon protein 1) (NOD26-like intrinsic protein 2-1) (OsNIP2;1) (Silicon influx transporter LSI1)	NIP2-1 LSI1 SIIT1 Os02g0745100 LOC_Os02g51110 OJ1118_G04.16 OJ1734_E02.43 OsJ_008085	*Oryza sativa* subsp. *japonica* (Rice)	298	Ma et al, 2006
Q10SY9	Silicon efflux transporter LSI2 (Low silicon protein 2)	LSI2 SIET1 Os03g0107300 LOC_Os03g01700 OJ1384D03.1 OsJ_09099	*Oryza sativa* subsp. *japonica* (Rice)	472	Ma et al, 2007
Q67WJ8	Aquaporin NIP2-2 (Low silicon protein 6) (NOD26-like intrinsic protein 2-2) (OsNIP2;2)	NIP2-2 LSI6 Os06g0228200 LOC_Os06g12310 OsJ_019836 P0425F05.28-1	*Oryza sativa* subsp. *japonica* (Rice)	298	Yamaji et al 2008
Q9AV23	Silicon efflux transporter LSI3 (Low silicon protein 3)	LSI3 Os10g0547500 LOC_Os10g39980 OSJNBa0001O14.19	*Oryza sativa* subsp. *japonica* (Rice)	485	Yamaji et al, 2015
A9XG44	Low silicon 1 protein (NOD26-like major intrinsic protein)	NIP2-1 Lsi1 Sb04g028020 SORBI_3004G238100	*Sorghum bicolor* (Sorghum) (*Sorghum vulgare*)	297	
G0WXH5	Silicon transporter protein	TaLsi1	*Triticum aestivum* (Wheat)	295	Montpetit et al 2012
B9X078	NIP2;1 (NOD26-like intrinsic protein) (Silicon transporter)	HvLsi1 HvNIP2;2 NIP2;1	*Hordeum vulgare* (Barley)	295	Chiba et al, 2009
Q19KC1	Aquaporin NIP2-1 (NOD26-like intrinsic protein 2-1) (ZmNIP2-1)	NIP2-1 LSI1	*Zea mays* (Maize)	295	Mitani et al, 2009

	(ZmNIP2;1)				
F1SX52	Silicon efflux transporter CmLsi2-1	CmLsi2-1	*Cucurbita moschata*	549	
F1SX53	Silicon efflux transporter CmLsi2-2	CmLsi2-2	*Cucurbita moschata*	529	
C6KYS1	Silicon transporter	HvLsi6	*Hordeum vulgare* (Barley)	300	Yamaji et al, 2012
F1SX51	Silicon transporter 1	CmLsi1(B-)	*Cucurbita moschata*	288	Mitani et al, 2011
L0P128	Low silicon transporter 2	lsi2	*Oryza sativa* subsp. *indica* (Rice)	472	
A0A172B0F5	Silicon transporter 3	Lsi3	*Cucurbita ficifolia* (figleaf gourd)	264	
A0A1S3ULW3	silicon efflux transporter LSI2	LOC106766708	*Vigna radiata* var. *radiata* (Mung bean) (*Phaseolus aureus*)	541	
A0A1S2Z0F2	silicon efflux transporter LSI2	LOC101491203	*Cicer arietinum* (Chickpea)	540	
Q1RU51	Silicon efflux transporter (Transporter, putative)	11444088 MTR_8g038580 MtrDRAFT_AC153125g34v2	*Medicago truncatula*	544	
A0A438E2S3	Silicon efflux transporter LSI2	LSI2_6 LSI2_2 CK203_019490 CK203_092880	*Vitis vinifera* (Grape)	471	
A0A1S3UXS9	silicon efflux transporter LSI2	LOC106769659	*Vigna radiata* var. *radiata* (Mung bean) (*Phaseolus aureus*)	531	
A9XHW6	NOD26-like major intrinsic protein	NIP2-2 SORBI_3010G092600	*Sorghum bicolor* (Sorghum) (*Sorghum vulgare*)	295	
A0A072U9P9	Silicon efflux transporter	25496368 MTR_6g045483	*Medicago truncatula* (Barrel medic) (*Medicago tribuloides*)	453	
A0A2H3Z9C2	silicon efflux transporter LSI2-like isoform X1	LOC103721631	*Phoenix dactylifera* (Date palm)	553	
B2ZHM7	Silicon transport protein (Fragment)		*Cenchrus americanus* (Pearl millet) (*Pennisetum glaucum*)	187	
A0A2Z2GWX9	Silicon transporter (Fragment)	Lsi2	*Lolium perenne* (Perennial ryegrass)	317	Pontigo et al, 2017
A0A2Z2GYA0	Silicon transporter	Lsi1	*Lolium perenne* (Perennial ryegrass)	295	Pontigo et al, 2017
A0A2H3YGM2	silicon efflux transporter LSI2-like	LOC103713336	*Phoenix dactylifera* (Date palm)	512	

A0A2H3YR70	aquaporin NIP2-1-like	LOC103716402	*Phoenix dactylifera* (Date palm)	290	Bokor et al., 2019
A0A2K3NKB6	Silicon efflux transporter	L195_g026824	*Trifolium pratense* (Red clover)	502	
A0A2K3LBI2	Silicon efflux transporter	L195_g031849	*Trifolium pratense* (Red clover)	476	
A0A2K3KWS9	Silicon efflux transporter (Fragment)	L195_g057699	*Trifolium pratense* (Red clover)	179	
A0A1S3UQ30	silicon efflux transporter LSI2-like	LOC106767626	*Vigna radiata* var. *radiata* (Mung bean) (*Phaseolus aureus*)	536	
A0A2H3X6R1	silicon efflux transporter LSI2-like	LOC103698921	*Phoenix dactylifera* (Date palm)	538	
A0A2H3Y7B5	silicon efflux transporter LSI2-like	LOC103710354	*Phoenix dactylifera* (Date palm)	543	
A0A072U8Q6	Silicon efflux transporter	25496368 MTR_6g045483	*Medicago truncatula* (Barrel medic) (*Medicago tribuloides*)	388	
A0A072UJZ4	Silicon efflux transporter	25496368 MTR_6g045483	*Medicago truncatula* (Barrel medic) (*Medicago tribuloides*)	465	
A0A1D6JKD4	Silicon transporter	100502546 ZEAMMB73_Zm00001d027305	*Zea mays* (Maize)	189	
A0A0B2PIX5	Putative transporter arsB (Silicon efflux transporter LSI3)	D0Y65_044088 glysoja_029087	*Glycine soja* (Wild soybean)	536	
A0A0B2PNP3	Putative transporter arsB (Silicon efflux transporter)	D0Y65_044086 glysoja_029085	*Glycine soja* (Wild soybean)	542	
G8XUV3	Si transport-like protein 1 (Silicon transporter Lsi1)	Csa_3G826640	*Cucumis sativus* (Cucumber)	288	
A0A445GK82	Silicon efflux transporter LSI3 isoform A (Silicon	D0Y65_044087	*Glycine soja* (Wild soybean)	538	
A0A0B2QK41	efflux transporter LSI3 isoform B)			534	
A0A445IRK1	Silicon efflux transporter LSI3	D0Y65_027904	*Glycine soja* (Wild soybean)	399	
C7G3B4	Silicon transporter	ZmLsi2 100502546 ZEAMMB73_Zm00001d027305	*Zea mays* (Maize)	477	Mitani et al, 2009
	Putative transporter arsB (Silicon efflux transporter	D0Y65_027904 glysoja_027655	*Glycine soja* (Wild soybean)		

	LSI3 isoform A) (Silicon efflux transporter LSI3 isoform B) (Silicon efflux transporter LSI3 isoform C) (Silicon efflux transporter LSI3 isoform D) (Silicon efflux transporter LSI3 isoform E)				
A0A0B2S8R6	Putative transporter arsB (Silicon efflux transporter LSI3 isoform A) (Silicon efflux transporter LSI3 isoform B)	D0Y65_024076 glysoja_038333	*Glycine soja* (Wild soybean)	523	
A0A445J0J3	Silicon efflux transporter LSI3 isoform A (Silicon efflux transporter LSI3 isoform B) (Silicon efflux transporter LSI3 isoform C) (Silicon efflux transporter LSI3 isoform D)	D0Y65_024075	*Glycine soja* (Wild soybean)	538	
A0A445IRM1	Silicon efflux transporter LSI3 isoform F (Silicon efflux transporter LSI3 isoform G) (Silicon efflux transporter LSI3 isoform H) (Silicon efflux transporter LSI3 isoform I) (Silicon efflux transporter LSI3 isoform J)	D0Y65_027904	*Glycine soja* (Wild soybean)	449	
A0A172B7P2	Silicon transporter 3	Lsi3	*Cucurbita moschata*	264	
F1SX50	Silicon transporter 1	CmLsi1(B+)	*Cucurbita moschata*	288	Mitani et al., 2011
B9T848	Silicon transporter,	RCOM_0100750	*Ricinus communis* (Castor bean)	297	

	putative				
I4IY30	aquaporin silicon transporter	EaNIP3,1	*Equisetum arvense* (Field horsetail) (Common horsetail)	248	Gregoire et al, 2012
I4IY32	aquaporin silicon transporter	EaNIP3,3	*Equisetum arvense* (Field horsetail) (Common horsetail)	259	Gregoire et al, 2012
I4IY33	aquaporin silicon transporter	EaNIP3,4a	*Equisetum arvense* (Field horsetail) (Common horsetail)	260	Gregoire et al, 2012
XP_004240725.1	Si influx transporter (active)	SILs2-L1	*Solanum esculentum* (Tomato)	528	Sun et al., 2019
XP_010317628.1	Si efflux transporter (inactive)	SILs2_L2	*Solanum esculentum* (Tomato)	516	Sun et al., 2019
